# Do plant biostimulants affect the survival of *Escherichia coli* in lettuce?

**DOI:** 10.3389/fpls.2024.1495463

**Published:** 2024-12-17

**Authors:** Leonardo Fiore, Mariateresa Cardarelli, Maurizio Ruzzi, Anna Grazia Ficca, Youssef Rouphael, Francesca Luziatelli, Giuseppe Colla

**Affiliations:** ^1^ Department of Agriculture and Forest Sciences, University of Tuscia, Viterbo, Italy; ^2^ Department for Innovation in Biological, Agrofood and Forest Systems, University of Tuscia, Viterbo, Italy; ^3^ Department of Agricultural Sciences, University of Naples Federico II, Portici, Italy

**Keywords:** *Escherichia coli*, biostimulant, lettuce, food safety, seaweed extract, protein hydrolysate, floating system, greenhouse

## Abstract

**Introduction:**

Considering that plant biostimulants can be sprayed multiple times on leafy crops even just before harvest, it is relevant to know the impact of biostimulant applications on *Escherichia coli* population dynamics of lettuce leaves to ensure food safety. Two trials were carried out to investigate whether the applications of a seaweed extract and a vegetal-derived protein hydrolysate (PH) could affect the *E. coli* growth in shake flasks (Exp. 1) and plant growth and survival of artificially inoculated *E. coli* on the leaf surface of lettuce grown in a floating system (Exp. 2).

**Methods:**

The non-pathogenic *E. coli* strain K12 was used in both trials. In Exp. 1, biostimulants’ inhibitory/stimulatory effect on *E. coli* growth was evaluated in liquid culture after 24 hours of incubation at 37°C. The 31-day agronomic trial (Exp. 2) was conducted in a polyethylene greenhouse on lettuce grown in a floating system.

**Results:**

In Exp. 1, *E. coli* growth was not affected by LB medium amended with biostimulants, whereas both biostimulants stimulated total aerobic bacteria and inhibited *E. coli* population on lettuce leaves with a more pronounced inhibitory effect of PH applications on *E. coli* (Exp. 2). Total plant biomass and its partitioning (on fresh and dry weight basis), and N concentrations (as total N and nitrates) of leaves were not influenced by both biostimulant treatments.

**Conclusion:**

The use of plant biostimulants could be a valuable and sustainable strategy to improve the microbiological quality of leafy greens to produce ready-to-eat foods.

## 1 Introduction

The minimally processed ready-to-eat leafy vegetables market has rapidly grown in the last decade, driven by increasing consumer demand for convenient and healthy food (Market Research Reports, Industry Trends & Analysis, 2024). The quality-defining parameters of this category of products are lack of defects, consistent organoleptic characteristics, high nutritional value, and hygienic safety (Colelli and Elia, 2009). Hygienic safety is related to inherent antinutritional substances, such as nitrate and oxalate, which accumulate during growth, and microbial contamination, particularly relevant for leafy green vegetables with high water content (Hackl et al., 2017). Microbial contamination of fresh-cut produce may occur from farm to table and pose threats to human health by causing various diseases like diarrhea, abdominal cramps, vomiting as well as death. Studies conducted over the past few years demonstrated that there was a correlation between the increase in outbreaks of foodborne illness due to *Escherichia coli* O157:H7, *Salmonella*, and *Listeria monocytogenes*, and fresh produce consumption (Slayton et al., 2013; Aiyedun et al., 2021). *E. coli* O157:H7 has survived and grown in a wide range of processed fruits and vegetables, such as lettuce (Abdul-Raouf et al., 1993; Abadias et al., 2012). *E. coli* reaches the edible product mainly through organic fertilizers (e.g., animal manure), irrigation, and processing water (Jensen et al., 2013; Belias et al., 2020; Nahim-Granados et al., 2021; Summerlin et al., 2021) and contaminated surfaces in processing environments (Ruzi et al., 2022; Sharma et al., 2022; Liu et al., 2023). Moreover, farmers can affect the *E. coli* population dynamics of the contaminated plant tissues by applying agricultural inputs such as fungicides and herbicides to the crops (Staley et al., 2014). Farmers are spraying crops more and more frequently with plant biostimulants to improve crop resistance to abiotic stresses, nutrient use efficiency, and quality traits (Cardarelli et al., 2024). Seaweed extracts from *Ascophyllum nodosum* and vegetal-derived protein hydrolysates are the most common plant biostimulants used as foliar spray, especially in vegetable crops. Previous studies demonstrated that foliar applications of a commercial vegetal-derived protein hydrolysate can change microbial community structure, stimulating the growth of epiphytic bacteria with plant growth-promoting and biological control activity against plant pathogens (Cappelletti et al., 2016; Luziatelli et al., 2019). However, more information is needed on the influence of seaweed extracts and protein hydrolysates on the population dynamics of the human pathogen *E. coli* in vegetable crops. Considering that plant biostimulants can be sprayed multiple times on leafy crops even just before harvest, it is relevant to know the impact of biostimulant applications on *E. coli* population dynamics of lettuce leaves to ensure food safety. Starting from the above considerations, two trials were carried out to investigate whether the applications of two widely used plant biostimulants, such as an *A. nodosum* seaweed extract and a vegetal-derived protein hydrolysate, could affect *E. coli* growth in shake flasks (Exp. 1), and survival of artificially inoculated *E. coli* on the leaf surface of lettuce grown in a floating system (Exp. 2). Moreover, the effects of biostimulants on growth and quality traits of lettuce plants were also evaluated in experiment 2.

## 2 Materials and methods

### 2.1 Bacterial strain and culture conditions

The non-pathogenic *E. coli* strain K12 (EC1; Merck KGaA, Darmstadt, Germany) was selected as a surrogate for *E. coli* O157:H7 due to its similar growth characteristics. LB Lennox (Difco BD Biosciences; Lennox, 1955) was used to activate lyophilized culture and its routine culturing in LB broth. Cultivation was performed in Erlenmeyer flasks at 37°C and 180 rpm. The strain was stock frozen using LB broth containing glycerol 20% (*vol/vol*).

### 2.2 Biostimulant characteristics

A commercial vegetal-derived protein hydrolysate and a commercial seaweed extract were tested in the trials. The vegetal-derived protein hydrolysate (PH) biostimulant Trainer^®^ was provided by Hello Nature, Rivoli Veronese, Italy. The PH biostimulant is obtained through enzymatic hydrolysis of legume seeds, and it contains 310 g/kg of free amino acids and peptides. The product’s detailed aminogram and other components were reported in detail by Rouphael et al. (2017) and Paul et al. (2019). The seaweed extract (SWE) biostimulant ‘Toggle^®^’, produced by Acadian Plant Health, Nova Scotia, Canada, is derived and refined from North Atlantic *Ascophyllum nodosum* sources. SWE contains 20 g/kg of organic carbon and 7 g/kg of mannitol.

### 2.3 Growth conditions of *E. coli* and experimental treatments in the shake flasks experiment

The inhibitory/stimulatory effect of biostimulants on *E. coli* strain K12 growth was evaluated in a liquid culture on an LB medium amended with 20% (vol/vol) filter sterilized biostimulants ‘Trainer^®^’ or ‘Toggle^®^.’ The sterilization was carried out using cellulose acetate sterile syringe filters (0.2µm) to remove vegetative cells of microorganisms that could be present in the commercial biostimulants, for which, according to EU 2019/1009, no limits are required for aerobic/anaerobic bacteria rather than *Salmonella*, *E. coli*, and *Enterococcaceae*.

An overnight preculture was prepared by inoculating 50 mL of LB medium with 0.5 mL of glycerol stock and grown at 37°C and 180 rpm. This culture was used to inoculate a 500 mL flask containing 50 mL of LB supplemented with the biostimulants (20% *vol/vol*), with an initial optical density (OD_600_) of 0.1. After 24 hours of growth, the culture was appropriately diluted, and aliquots (0.1 mL) were spread onto LB agar plates. The latter were incubated for 24 hours at 37°C before colony counting.

### 2.4 Plant growth conditions in the greenhouse experiment

The agronomic trial was conducted in summer 2023 in a tunnel greenhouse of 200 m^2^ (25m *8 m) having a volume/surface ratio of 2.6 and covered with polyethylene film of thickness equal to 0.2 mm at the Experimental Farm of Tuscia University, central Italy (latitude 42° 25’ N, longitude 12° 08’, altitude 310 m). Plants were grown under natural light conditions. During the growing cycle, the average daily mean, minimum and maximum air temperature was 26, 18, and 34°C respectively. The average daily mean, minimum and maximum air humidity was 62, 48, and 80%, respectively. Lettuce seeds (*Lactuca sativa* L. var. *acephala* cv. Green Salad Bowl; SAIS S.p.A., Cesena, Italy) were sown on May 26 in polystyrene trays filled with vermiculite to assure a final plant density of 27 plants per tray. Polystyrene trays were placed in black plastic tanks (0.35x0.25 m; 0.14 m height) filled with 8 L of nutrient solution each. The nutrient solution was obtained by adding the following fertilizers to pure water (mg/L): 722 Ca(NO_3_)_2_, 136 KH_2_PO_4_, 182 K_2_SO_4_, 203 KNO_3_, 384 Mg(NO_3_)_2_, 81 mM NH_4_NO_3_. Micronutrients were added as 24 mg/L of commercial fertilizer (Mikrom; Cifo S.p.A., Bologna, Italy) containing (g/kg): 5 B, 5 Cu, 40 Fe, 40 Mn, 2 Mo, 10 Zn, 18 Mg, 24 S. The volume of the nutrient solution was kept constant at 8 L, and oxygenation of nutrient solution was conducted by pumping air through emitters placed on the bottom of the tanks (one emitter for tank); the oxygenation of the nutrient solution was managed for assuring a dissolved oxygen concentration above 6 mg/L. During the growing cycle, nutrient solutions’ electrical conductivity and pH were 2.0 ± 0.2 dS m^-1^ and 6.0 ± 0.3, respectively.

### 2.5 Preparation of *E. coli* suspension for plant inoculation

To prepare the *E. coli* strain K12 suspension, 10 mL of an overnight preculture, as described earlier, was used to inoculate the working culture (100 mL LB medium). The culture was then allowed to grow for 18 hours at 37°C and 180 rpm. After this time, the culture was diluted 10^3^-fold in sterile water to obtain a concentration of approximately 10^6^ viable cells per mL (determined by Most Probable Number technique), which was used for plant inoculation. All experiments were carried out in triplicates and the results were expressed as the mean ± standard deviation.

### 2.6 Treatments, plant inoculation, and recovery of *E. coli* cells in the greenhouse experiment

The trial included the following treatments: foliar applications of the seaweed extract (SWE) ‘Toggle^®^’ at 2 g/L, foliar applications of the protein hydrolysate (PH) ‘Trainer^®^’ at 3 g/L, and untreated control. Biostimulants were applied twice at 18^th^ and 25^th^ days after sowing (DAS) corresponding to BBCH 13 and BBCH 15, respectively. An untreated control was foliar sprayed with pure water on the same day as the biostimulant applications. The volume of biostimulant solution/water for each treatment was 15 ml/plot. Treatments were arranged in a randomized block design with four replicates per treatment (1 tank as an experimental unit). On 16^th^ DAS (BBCH 13), *E. coli* suspension, prepared as previously described, was sprayed at a rate of 20 ml/plot on the leaves to simulate an *E. coli* contamination of the lettuce crop before biostimulant application. One day before (15^th^ DAS), and one day after (17^th^ DAS), the foliar application of *E. coli* suspension, and two days after each biostimulant treatment (20^th^ and 27^th^ DAS), four lettuce plants per treatment were harvested. The outermost leaves of the lettuce shoots were aseptically removed, and four inner leaves per plant were aseptically cut, carefully bagged, transported to the laboratory, and processed within 24 hours. Leaf samples of each treatment were homogenized in a sterile plastic bag with 20 mL of 1% buffered peptone water (*w/vol*) using a stomacher (BagMixer 400S; Interscience, France). The resultant supernatant was appropriated, diluted with 1% buffered peptone water, and seeded (0.1 mL) onto LB and McConkey agar plates. LB plates were incubated at 30°C for 48 hours for counting of predominant aerobic bacteria, while McConkey agar plates were incubated at 44°C for 24 hours, and typical pinkish colonies, indicative of lactose fermenting bacteria, were counted for enumeration of *E. coli* strain K12.

**Figure 1:**
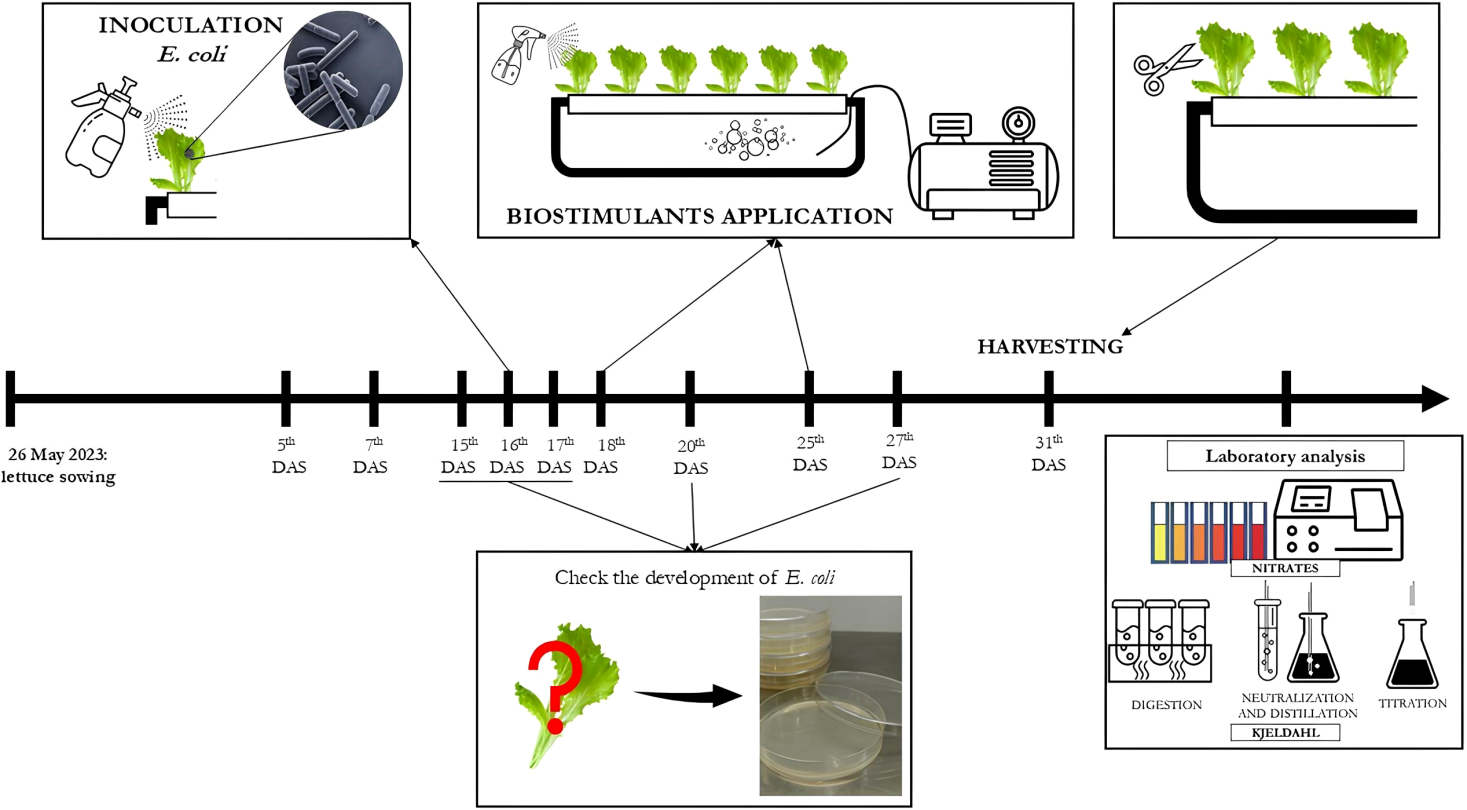
Timeline of treatment applications, *E. coli* inoculation, and measurements and analysis in the lettuce trial.

**Figure 1** reports a timeline of the treatments, *E. coli* suspension application, measurements, and analysis performed during the greenhouse lettuce trial.

### 2.7 Plant biomass, nitrogen, and nitrate concentration in lettuce leaves

On the 31^st^ DAS, lettuce plants were harvested to record the fresh weight of shoots and roots. Shoot and root dry weight were determined after oven-drying plant tissues at 65°C until the sample weight remained constant. Dry leaf samples were ground separately in a Wiley mill to pass through a 20-mesh screen, and then 0.5 g of the dried plant tissues were used to determine nitrates and total Kjeldahl nitrogen. For nitrate determination, the 5% salicylic acid method was used (Vendrell and Zupancic, 1990); briefly, nitrates were extracted from plant tissues after mixing and centrifugation at room temperature using distilled water as the extractant and the reading were performed spectrophotometrically at 410 nm. Nitrogen concentration in the leaf tissues was determined after mineralization with sulfuric acid (96%), distillation of the NH_3_ produced and its quantification by titration with HCl 0.1 N (‘Kjeldahl method’ -Anderson and Ingram, 1990).

### 2.8 Statistical analysis

Analysis of variance (ANOVA) of the data was performed using GraphPad Prism version 8.0.1. Before ANOVA, the experimental data were checked for normal distribution and homogeneity of variance using Levene’s test. Tukey’s test was carried out at p = 0.05 on each of the significant variables measured.

## 3 Results

### 3.1 Effect of biostimulants on *E coli* growth in shaken flasks

In the laboratory experiments, the effect of biostimulants on *E. coli* strain K12 growth was evaluated in a shaken flask trial carried out on an LB medium amended with SWE and PH. The inhibitory/stimulatory effect was assessed by comparing the viable culturable cells of overnight culture grown with and without the biostimulant applications, measured as Colony Forming Units (CFU) per mL. Results indicated that neither of the two biostimulants statistically affected the *E. coli* growth compared to the untreated control. The mean cell density values of *E. coli* K12 ± standard errors were as follows (CFU/ml): 3.2 ± 0.5 *10^8^ for untreated control; 3.4 ± 0.2 *10^8^ for SWE; 2.9 ± 0.6 x10^8^ for PH.

### 3.2 Effects of biostimulants on plant biomass, nitrogen, and nitrate concentration in lettuce leaves

No significant differences were observed among treatments for shoot, root, and total biomass of lettuce plants on fresh and dry weight basis (**Table 1**). Similarly, no significant differences were recorded between nitrate treatments and total nitrogen concentration in lettuce leaves (**Table 1**).

**Table 1:** Effects of biostimulant treatments on lettuce biomass and nitrate and total nitrogen concentration in lettuce leaves.

Treatments	Fresh biomass (kg/m^2^)			Dry biomass (g/m^2^)			Nitrates (mg NO_3_/kg f.wt.)	Kjeldahl Nitrogen (g/kg d. wt.)
	Shoots	Roots	Total	Shoot	Root	Total		
Control	4.05 ± 0.24	0.63 ± 0.07	4.69 ± 1.71	237.8 ± 16.3	31.6 ± 1.3	269.4 ± 103.1	2991.4 ± 151.1	43.8 ± 0.5
SWE	4.03 ± 0.27	0.70 ± 0.04	4.73 ± 1.67	233.2 ± 25.1	31.6 ± 0.8	264.9 ± 100.8	3223.5 ± 230.9	43.4 ± 0.5
PH	4.34 ± 0.41	0.63 ± 0.05	4.98 ± 1.85	242.8 ± 18.0	31.2 ± 0.5	274.0 ± 105.8	3262.0 ± 84.5	44.7 ± 0.8
Significance	ns	ns	ns	ns	ns	ns	ns	ns

SWE, seaweed extract ‘Toggle^®^’; PH, protein hydrolysate ‘Trainer^®^’. ns, not significant according to Tukey’s test (p > 0.05).

### 3.3 Effect of biostimulants on *E coli* growth on lettuce leaves

Leaves from treated and untreated plants were collected at different times to study the effects of biostimulant treatments on the abundance of the total aerobic cultivable bacterial and *E. coli* population (**Figure 1**). One day before *E. coli* strain K12 inoculation (15 DAS), the average total population of aerobic cultivable bacteria in lettuce leaves across treatments was 1.58*10^2^ CFU/g biomass, while *E. coli* was not detected in the lettuce leaves from all plots. After biostimulant applications, the data reported in **Table 2** indicated that the abundance of aerobic bacteria did not significantly change between the 17^th^ DAS and 27^th^ DAS in untreated plants. In both biostimulant-treated plants, a significant increase in the total aerobic population was observed two days after the first treatment (20^th^ DAS). This effect was more pronounced with SWE than PH: 6*10^3^-fold and 3.9-fold compared to untreated plants (**Table 2**). On the 20th and 27th DAS, biostimulant-treated plants’ total aerobic bacterial population was significantly higher than untreated plants, especially in SWE-treated leaves. The leaf inoculation with a suspension containing *E. coli* strain K12 effectively raised the leaf *E. coli* population one day after leaf inoculation (17 DAS, **Table 2**). On the following sampling dates (20 and 27 DAS), a decrease in the *E. coli* population was recorded in all treatments (**Table 2**). This effect was more pronounced in PH-treated plants where the abundance of *E. coli* was already below the detection limit after the first biostimulant treatment (20 DAS). A similar effect was achieved with SWE only after the second biostimulant treatment (27 DAS). In untreated plants inoculated with the *E. coli* K12 strain, the presence of *E. coli* was detected until the end of the experiment (**Table 2**).

**Table 2:** Effect of biostimulant treatments on the total epiphytic aerobic bacterial and *E. coli* population of lettuce leaves.

Treatments	Total aerobic bacteria (CFU/g biomass^1^)			*E. coli* (CFU/g biomass^1^)		
	17 DAS	20 DAS	27 DAS	17 DAS	20 DAS	27 DAS
Control	1.4 ± 0.6*10^2^ a	1.4 ± 0.3*10^2^ c	1.3 ± 0.4*10^2^ c	4.0 ± 0.3*10^4^ a	63.1 ± 2.3 a	22.8 ± 6.7 a
SWE	1.4 ± 0.6*10^2^ a	9.0 ± 0.5*10^5^ a	4.6 ± 0.8*10^2^ a	4.0 ± 0.3*10^4^ a	9.2 ± 0.1 b	0.0 b
PH	1.4 ± 0.6*10^2^ a	5.5 ± 0.2*10^2^ b	3.1 ± 0.5*10^2^ b	4.0 ± 0.3*10^4^ a	0.0 c	0.0 b
Significance	ns	**	*	ns	**	**

SWE, seaweed extract ‘Toggle^®^’; PH, protein hydrolysate ‘Trainer^®^’. DAS, days after sowing. ^ns,*,**^ not significant or significant according to Tukey’s test for p<0.05 and p<0.01, respectively. ^1^Viable cell density measured by plating appropriate dilutions of leaf microbiome on LB (Total aerobic bacteria) and McConkey agar (*E. coli* K12) plates.

## 4 Discussion

*E. coli* is not harmful to plants, but some *E. coli* strains are well-known as human pathogens. Enterohemorrhagic *E. coli* (EHEC) is a foodborne pathogen and is responsible for several global outbreaks related to the consumption of contaminated fresh-cut products (Bottichio et al., 2020; Coulombe et al., 2020; Irvin et al., 2021). In the shake flask experiment (Exp. 1), the *E. coli* K12 strain population was not significantly changed from applying both biostimulants, indicating the lack of direct effects of biostimulant components on *E. coli* K12 strain growth. The above findings may be explained by the presence of sufficient amounts of nutritional compounds in the LB medium for supporting optimal growth of the *E. coli* K12 strain. Moreover, it is interesting to note that both biostimulants did not contain inhibitory compounds for the growth of the *E. coli* K12 strain.

In many experimental studies, plant biostimulants have been reported to increase yield, nutrient use efficiency, and quality of greenhouse leafy vegetables such as lettuce, spinach, and basil (Rouphael et al., 2018; Carillo et al., 2019; Ciriello et al., 2022). The biostimulant effects of PH have been attributed to bioactive compounds like peptides and amino acids exerting auxin and gibberellin-like activity (Colla et al., 2014), while polysaccharides, phenolic compounds, and osmolytes (proline, betaine, and mannitol) have been reported to be the most active compounds in SWE (Sharma et al., 2014). Contrary to the above research findings, no significant effects of both biostimulant applications on lettuce biomass and its partitioning were found in the current experiment (Exp. 2). As reported in several studies (Vernieri et al., 2006; Carillo et al., 2019) the biostimulant effects of PH and SWE are affected by the fertilization regime of leafy vegetables. For instance, Carillo et al. (2019) reported that the yield increase of greenhouse spinach resulting from foliar PH applications was significant only at lower nitrogen fertilization rates (0 and 15 kg N/ha), while there were no significant differences in shoot yield under high N supply (30 and 45 kg N/ha). Similarly, Vernieri et al. (2006) observed that the biostimulant-mediated yield increase of hydroponically grown rocket at different nutrient solution concentrations (standard Hoagland solution concentration; 4-fold diluted solution; 10-fold diluted solution) was significant only under the lowest nutrient solution concentration. The above findings may explain the lack of significant effects of PH and SWE on lettuce biomass in our experimental conditions where nutrients were supplied to lettuce crops at optimal concentrations. The optimal nutritional status of lettuce crop was also confirmed by the high levels of total nitrogen recorded in leaf tissues from all treatments at the end of the experiment; in the current trial, the average N concentration in lettuce leaves was 44 g N/kg, which was within the optimal N range (33- 48 g N/kg) reported by Hartz et al. (2007) for lettuce crop. Timing and method (root or foliar application) of biostimulant application may also have affected the crop growth response under a floating system, as reported in several studies (Cristofano et al., 2021). Moreover, nitrate assimilation was not improved in biostimulant-treated leaves, as demonstrated by the lack of significant differences among treatments for total nitrogen and nitrate concentrations in lettuce leaves (**Table 1**). The average nitrate concentration (3159 mg NO_3_/kg FW) was below the maximum level (4000 mg NO_3_/kg FW) set by EC Regulation 1258/2011 for the marketability of fresh lettuce in the European Union. In agreement with our findings, Rouphael et al. (2018) reported that leaf nitrate concentration of greenhouse spinach was not increased by foliar applications of PH ‘Trainer^®^’ in comparison with untreated control; however, in the same greenhouse spinach trial, foliar applications of two other biostimulants (a seaweed extract from *Echlonia maxima* and a vegetal biostimulant) significantly increased the leaf nitrate concentrations in comparison with untreated control highlighting the importance of a careful evaluation of biostimulant effects on nitrogen metabolism for assuring high quality of leafy vegetables.

Foliar applications of the two biostimulants affected the bacterial microbiome and significantly increased the total aerobic bacterial population (**Table 2**). This effect was powerful in SWE-treated plants (20 DAS), indicating that different biostimulant products affect differently the plant holobiome. These findings are in agreement with Luziatelli et al. (2019), who demonstrated that using the vegetal-derived protein hydrolyzate Trainer^®^ enhances microbiome diversity in lettuce plants and favors the growth of specific taxa. In the current study, a decrease in the total aerobic bacterial population at 27 DAS (two days after the second biostimulant application) was recorded in lettuce leaves of both biostimulant treatments. In contrast, the total aerobic bacterial population in the untreated control did not significantly change during the trial (**Table 2**). The above findings may be related to the positive effects of organic compounds (e.g., amino acids, sugars) provided by the biostimulant applications in supporting microbial growth. Interestingly, viable *E. coli* cells in untreated leaves were detectable for the entire period of the trial, although their concentration decreased to 22.8 CFU/g at the end of the experiment. The latter value is five-fold lower than the lower *E. coli* guideline limits for fresh vegetables reported in EC 2073/2025 (100-1000 CFU/g). In the biostimulants-treated leaves, the decrease in the *E. coli* population was faster than in untreated leaves (**Table 2**). At 20 DAS, PH treatment caused the total inhibition of the *E. coli* population in lettuce leaves, while SWE treatment induced a reduction of the *E. coli* population, which was 7-fold lower than untreated leaves (**Table 2**). At 27 DAS, the *E. coli* population was not detected in the leaves of both biostimulant treatments. These findings indicate that under *in vivo* conditions, both biostimulants harm the viability of *E. coli* cells. Moreover, the lack of direct effects of biostimulants on *E. coli* in the shake flask experiment indicates that the significant differences in the E. coli population recorded in the lettuce trial were probably due to indirect effects of both biostimulants on *E. coli* (e.g., stimulation of epiphytic microorganisms which are antagonists to *E. coli*).

## 5 Conclusions

This is the first report in scientific literature demonstrating that plant biostimulants, while stimulating the growth of plant-associated aerobic bacteria, negatively affect *E. coli* viability in lettuce leaves. The use of plant biostimulants as plant growth-promoting agents could be a valuable and sustainable strategy to improve the microbiological quality of leafy greens to produce ready-to-eat foods. However, additional studies are necessary to evaluate the impact of different biostimulant doses and concentrations, and other types of plant biostimulants (e.g., seaweed extracts and protein hydrolysates from different sources, humic-derived biostimulants) on *E. coli* and understand the mechanisms behind the biostimulant effects on *E. coli* population in plant tissues.

## Data Availability

The raw data supporting the conclusions of this article will be made available by the authors, without undue reservation.
